# A Vehicular IoT-Based Methane Sensing System for Large-Scale Urban Environmental Monitoring

**DOI:** 10.3390/s26144491

**Published:** 2026-07-15

**Authors:** Nuncio Perrella, Fuad Kassab, Angelo Zanini

**Affiliations:** 1 Polytechnic School, University of Sao Paulo, Sao Paulo 05508-010, Brazil; fuad@lac.usp.br; 2 Maua Institute of Technology, Sao Caetano do Sul 09580-900, Brazil; angelo.zanini@maua.br

**Keywords:** methane sensor, Internet of Things (IoT), vehicular sensing, urban environmental monitoring, smart cities, mobile sensor networks, air pollution, greenhouse gases

## Abstract

This paper presents the design, development, and large-scale deployment of a vehicular IoT-based methane sensing system for urban environmental monitoring. The proposed solution integrates a low-cost metal oxide semiconductor (MOS) methane sensor with a dual chamber gas sampling mechanism, embedded processing, and wireless communication via 4G/5G networks using a smartphone as a gateway. Methane concentration data are collected from sensors installed in moving vehicles, georeferenced in real time using GNSS, and transmitted to a cloud-based platform for storage and analysis. Field experiments were conducted in the metropolitan region of São Paulo, Brazil, using 16 instrumented vehicles over a 20-month period, covering approximately 192,274 km and generating more than 48 million measurements. The results reveal spatially consistent methane concentration patterns and identify urban areas with elevated levels exceeding global background concentrations. A comparative analysis with a commercial infrared-based mobile methane monitoring system showed consistent agreement in the identification of spatial methane concentration patterns and potential emission hotspots. These results demonstrate the effectiveness of the proposed system for scalable urban methane monitoring.

## 1. Introduction

Atmospheric methane concentrations play a critical role in climate regulation and represent one of the most significant contributors to global warming due to their high global warming potential [1,2,3]. Climate change affects natural ecosystems and human society in multiple ways, including rising global temperatures, sea-level increase, reduced agricultural productivity, biodiversity loss, and adverse impacts on public health. In this context, the continuous monitoring of methane emissions has become essential for understanding emission sources, identifying spatial and temporal trends, and supporting mitigation strategies aimed at reducing environmental and societal impacts.

Although methane monitoring has traditionally focused on natural gas infrastructure, petroleum production, landfills, and agricultural activities, urban environments also contain multiple potential methane sources. Consequently, identifying persistent methane hotspots through large-scale mobile monitoring represents an important first step toward understanding urban methane emissions and supporting future source attribution and mitigation strategies.

Methane sensing systems are required to exhibit high reliability and robustness, typically integrating microprocessors, signal conditioning circuits, and sensing elements based on different technologies, such as electrochemical, non-dispersive infrared (NDIR), catalytic, and metal-oxide semiconductor (MOS) sensors [4]. Several studies have proposed fixed methane monitoring systems based on wireless sensor networks and IoT architectures [5,6,7]. For instance, the system described in [7] employs solar powered sensor nodes with 3G/4G communication, capable of detecting methane within a limited spatial radius of approximately one meter. Other approaches based on mobile sensing platforms and vehicular methane monitoring have also been reported in the literature [8].

While effective for localized monitoring, extending such an approach to cover an entire metropolitan area such as the city of São Paulo, which spans approximately 1523 km^2^, would require a dense network of sensor nodes, making large-scale deployment economically challenging and operationally complex.

To address these limitations, this work proposes a mobile methane monitoring approach based on sensors installed on moving vehicles. A key distinguishing feature of this work, in comparison with related methane sensing approaches [8], lies in the adoption of a dual-chamber gas retention architecture combined with an active air circulation pump. This design enables controlled gas sampling and retention, effectively compensating for the intrinsic response time of approximately 30 s associated with metal oxide semiconductor (MOS) sensors [9,10]. By alternating the operation between the two chambers, while one chamber performs measurement, the other accumulates the gas sample, the system ensures continuous acquisition without loss of spatial resolution. This approach allows reliable methane concentration measurements in mobile conditions at vehicle speeds below 50 km/h, overcoming a common limitation of low-cost MOS sensors in dynamic environments. As a result, the proposed system achieves an effective balance between performance, compactness, and cost, providing a scalable and practical solution for large-scale urban methane monitoring.

Beyond the development of a low-cost methane sensor, this study presents a monitoring ecosystem that integrates mobile sensing, real time geolocation, wireless communication, and cloud-based data storage and analysis. By leveraging vehicular mobility, the proposed system enables wide area urban methane monitoring using a significantly reduced number of sensors operating continuously across large geographic regions, offering a scalable and cost-effective alternative to traditional fixed monitoring infrastructures.

## 2. Sensor Selection and Comparative Analysis

The selection of gas sensing technology plays a critical role in the performance, reliability, and scalability of methane monitoring systems, particularly in mobile and urban environmental applications [11]. In this study, two distinct sensing approaches were evaluated: a metal oxide semiconductor (MOS) TGS2611-C00 methane sensor (Figaro Engineering Inc., Osaka, Japan) and an INIR-ME5% nondispersive infrared methane sensor (SGX Sensortech Ltd., Corcelles-Cormondrèche, Switzerland).

Although several methane sensing technologies are available, including catalytic, electrochemical, pyroelectric, and non-dispersive infrared (NDIR) sensors, the present study focuses exclusively on MOS and NDIR technologies for both technical and practical reasons. During the preliminary development phase, different sensing principles were experimentally evaluated with the objective of developing a low-cost, scalable, and robust mobile monitoring platform suitable for continuous operation under harsh urban conditions. Catalytic and pyroelectric sensors were investigated but did not provide a satisfactory balance between sensitivity, long-term stability, durability, power consumption, and implementation cost for the proposed application. Consequently, the MOS sensor was selected for the developed platform because it offered the best compromise between cost, robustness, ease of integration, and sensing performance. The NDIR technology [12,13] was selected as the reference because it is employed in the commercial Protheo Huberg (HGS SAS, Bolzano, Italy) [14] methane monitoring system installed on the vehicles used during the field validation campaign. Therefore, the comparison presented in this work is intended to evaluate the performance of the proposed low-cost MOS-based system against the NDIR-based commercial system currently used as the reference for mobile methane monitoring.

The TGS2611 C00 [10] is an MOS type gas sensor designed for methane detection, operating based on variations in sensor resistance in response to gas exposure. It offers advantages such as low cost, compact form factors, simple interface circuitry, and fast response characteristics. The sensor operates with a heater voltage of 5 V, requiring approximately 56 mA, and exhibits a power consumption of approximately 280 mW. However, due to its sensing principle, it exhibits cross sensitivity to other gases such as ethanol, hydrogen, and isobutane, and is influenced by environmental conditions such as temperature and humidity. In addition, sensor to sensor variability and long-term drift require periodic calibration and compensation strategies.

In contrast, the Integrated IR (INIR-ME5%) [15] sensor employs nondispersive infrared (NDIR) technology, enabling direct measurement of gas concentration through infrared absorption. The INIR integrates signal processing, temperature compensation, and calibration algorithms within the sensor module, providing both digital and analog outputs. The sensor demonstrates improved selectivity, reduced cross sensitivity, and enhanced long-term stability. Its typical response time (T90) ranges from 18 s to 30 s, with average current consumption below 32 mA. Additionally, the INIR-ME5% incorporates internal diagnostics, fault detection mechanisms, and automatic range switching, which are essential for safety critical and industrial applications.

From a system design perspective, the choice between MOS and NDIR technologies involves a tradeoff between cost, scalability, and measurement reliability. MOS sensors such as the TGS2611-C00 are well suited for high-density, low-cost deployments and applications where relative concentration changes are sufficient [16]. However, their limited selectivity and sensitivity to environmental variations may introduce uncertainties in quantitative methane measurements, particularly in complex urban atmospheres.

Conversely, NDIR sensors such as the INIR-ME5% provide superior metrological performance, including higher selectivity, stability, and direct concentration output, making them more suitable for applications requiring accurate quantification and compliance with environmental standards. Nevertheless, their higher cost and integration complexity may limit large scale deployment. Table 1 provides comparison between the MOS-based TGS2611-C00 sensor and the NDIR-based INIR-ME5% sensor using normalized performance metrics relevant to mobile methane monitoring applications. Table 1 highlights the complementary characteristics of both sensing technologies.

### Reference Methane Analyzer

To validate the proposed mobile sensing platform under real operating conditions, field measurements were compared with a commercial Protheo Compact methane analyzer.

The Protheo Compact is a vehicle-mounted methane leak detection system specifically designed for natural gas network inspection. The instrument employs an infrared (IR) methane analyzer operating with a dedicated optical measurement principle, providing selective methane detection without the cross-sensitivity typically associated with metal-oxide semiconductor sensors.

According to the manufacturer’s specifications, the analyzer provides a methane measurement resolution of 1 ppm and performs concentration measurements at 1 s intervals, allowing continuous acquisition during vehicle operation. Owing to its high selectivity and widespread use by natural gas distribution companies, the Protheo Compact was adopted as the reference instrument for the field validation experiments presented in this work.

It should be emphasized that the laboratory calibration of the proposed sensing platform was performed using the GT Series portable methane analyzer (Teledyne Gas and Flame Detection, Cypress, TX, USA) [17], whereas the Protheo Compact was employed exclusively as the reference instrument during the urban field campaigns. The purpose of the field comparison was not to evaluate the metrological accuracy of the Protheo Compact nor to calibrate the proposed sensing platform against the infrared analyzer. Laboratory calibration was performed independently using the GT Series reference analyzer under controlled conditions, as described in Section 9. The field experiments were intended solely to evaluate the capability of the proposed platform to reproduce the spatial distribution of methane concentrations observed by an established commercial mobile methane monitoring system.

## 3. Signal Processing

Accurate methane concentration estimation in mobile sensing systems requires appropriate signal processing strategies to account for sensor dynamics, environmental variability, and noise measurement. In this work, a time aligned sampling and filtering approach was implemented to ensure consistency between the sensor response characteristics and the data acquisition process. The gas sensing system operates with a cyclic sampling procedure consisting of two sequential stages: (i) a chamber filling phase and (ii) a gas measurement phase. Each stage has a duration of 1 s, resulting in a total acquisition period of 2 s per sample. This procedure allows controlled gas exchange within the sensing chamber while ensuring sufficient stabilization time before each measurement.

The sensor response time is commonly characterized by the T90 parameter, defined as the time required for the sensor to reach 90% of its final response following a step change in gas concentration. Considering the intrinsic response time of the sensor (T90 ≈ 30 s), individual measurements are subject to transient effects and short-term fluctuations. To mitigate these effects and improve measurement stability, a moving average filter was applied over a fixed window of 15 samples, corresponding to a total time window of 30 s. This design ensures temporal consistency between the acquisition window and the sensor response dynamics.

It should be emphasized that the 1 s measurement interval corresponds to the gas sampling cycle of the dual-chamber system rather than to the intrinsic response time of the TGS2611-C00 sensor. The sensing element remains continuously energized during operation and exhibits a typical T90 response time of approximately 30 s. Therefore, methane concentration is estimated from a moving-average window comprising 15 consecutive resistance measurements (30 s), followed by temperature–humidity compensation and nonlinear calibration, thereby ensuring stable and reliable concentration estimates.

Since the acquisition cycle produces one valid measurement every 2 s, the 15-sample moving-average window corresponds to a total processing window of 30 s. This duration was selected to match the typical $T_{90}$ response time of the TGS2611-C00 sensor. Therefore, the system does not require an additional 30 s stabilization period before averaging; instead, methane concentration is estimated continuously from the most recent 15 measurements, providing a filtered output that is temporally consistent with the intrinsic response dynamics of the MOS sensor.

The moving average is defined as

(1) $$\bar{x}(n)=\frac{1}{N}\sum_{i=0}^{N-1}x(n-i)$$

where *x*(*n*) represents the current gas concentration measurement and *N* = 15 is the number of samples in the averaging window. This filtering approach reduces high frequency noise and smooths transient variations, resulting in a more stable estimate of methane concentration.

To enable efficient real time implementation in embedded systems, the moving average was computed using a circular buffer structure. At each new measurement, the oldest sample is removed from the accumulated sum, and the newest sample is added, updating the average. This approach minimizes computational overhead and is well suited for microcontroller-based platforms.

The proposed sampling and filtering strategy is particularly suitable for mobile methane monitoring applications, where rapid spatial changes in gas concentration must be captured while maintaining measurement reliability. By aligning the averaging window with the sensor response time, the system effectively balances responsiveness and stability. It is important to note that while moving average filter improves signal stability, it introduces a smoothing effect that may reduce sensitivity to rapid concentration changes. However, given the relatively slow response dynamics of the sensing technology, this tradeoff is acceptable and consistent with the physical limitations of the sensor.

Overall, the adopted signal processing approach enhances the robustness of methane concentration measurements by reducing noise, compensating for transient effects, and ensuring temporal coherence with sensor behavior, thereby improving the quality and reliability of the collected data.

## 4. Gas Sampling System Design and Optimization

**Figure 1:**
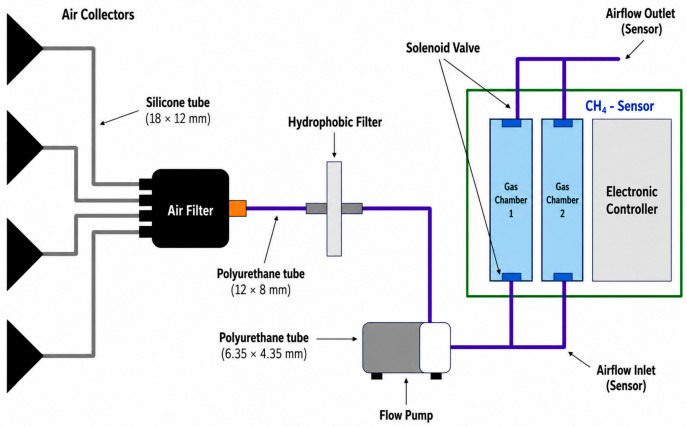
System Diagram.

The performance of methane sensing systems in mobile applications is strongly dependent on the efficiency and consistency of the gas sampling process [8,18]. An integrated gas sampling system was developed, comprising a controlled sampling chamber, a flow pump, and a valve-based flow control mechanism, as illustrated in Figure 1. The system was specifically designed to ensure rapid gas exchange, high measurement repeatability, and reliable operation under the dynamic conditions inherent to vehicular applications.

## 5. Chamber Dimensioning Based on Flow Rate

The sampling system employs a flow pump with a nominal flow rate of 3.2 L/min, corresponding to approximately 53.3 mL/s. The acquisition cycle was defined as 2 s, consisting of 1 s for chamber filling and 1 s for gas measurement. To ensure complete renewal of the gas within the chamber during the filling phase, chamber volume *V* was defined based on the volumetric flow rate *Q* and filling time t:

(2) V=Q⋅t

Using *Q* = 53.3 mL/s and *t* = 1 s, the resulting chamber volume is approximately 53.3 mL. The chamber was first implemented using cylindrical geometry for ease of integration and manufacturing. Considering a tube diameter of 21.5 mm, the chamber length L was calculated using:

(3) $$V=\pi r^{2}L$$

**Figure 2:**
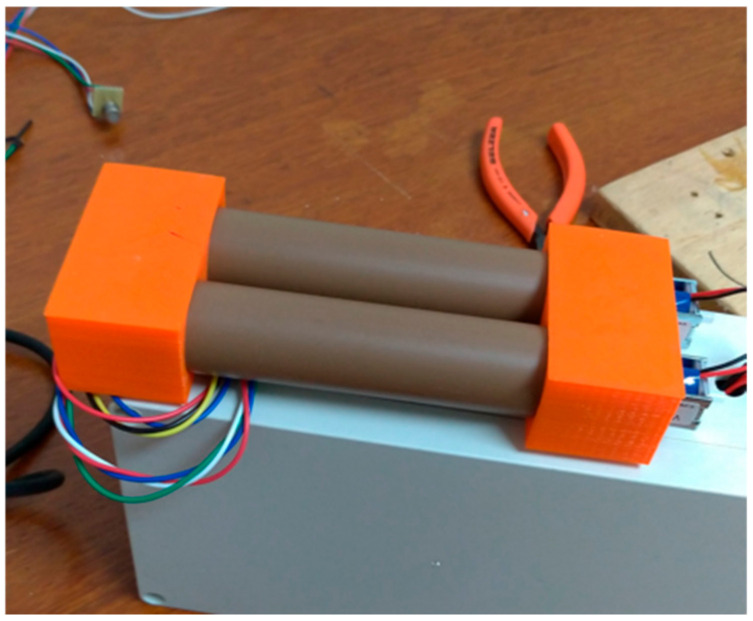
Chamber First Design.

Resulting in a length of approximately 147 mm. This design ensures that the chamber volume is properly matched to the pump capacity, enabling efficient gas exchange during each sampling cycle. Figure 2 illustrates the first chamber prototype.

## 6. Flow Control Using Pump and Valves

To improve system dynamics and measurement stability, an active gas flow approach was adopted [18]. The use of the flow pump enables forced gas intake and exhaust, significantly enhancing the response time and repeatability compared to passive diffusion. The system incorporates inlet and outlet valves to control the gas flow path. These valves enable sequential operation of the sampling cycle, including chamber filling, measurement, and purging phases. This controlled operation prevents residual gas from previous samples from affecting subsequent measurements and ensures that each acquisition cycle represents the current environmental conditions.

Experimental observations demonstrated that the inclusion of the flow pump substantially improved sensor response and signal stability, particularly under low airflow conditions or when the vehicle is stationary.

## 7. Chamber Design, Material Selection and CFD-Based Geometry Optimization

The gas sampling chamber was specifically designed to provide rapid gas exchange while maintaining mechanical robustness, low manufacturing cost, and compatibility with mobile deployment. The chambers were fabricated from Acrylonitrile Butadiene Styrene (ABS) using fused deposition modeling (FDM) 3D printing, allowing rapid prototyping and precise control of the internal geometry. ABS was selected because of its excellent mechanical properties, dimensional stability, and sufficient chemical inertness for atmospheric methane monitoring. Since methane is a chemically stable and non-polar molecule, its interaction with ABS surfaces is negligible under the operating conditions employed in this work. Furthermore, the continuous airflow generated by the sampling pump prevents residual gas accumulation within the chamber, minimizing adsorption effects and ensuring repeatable measurements during long-term operation. Once the chamber material and internal volume were defined, computational fluid dynamics (CFD) simulations were performed to optimize the internal geometry and improve gas exchange efficiency.

**Figure 3:**
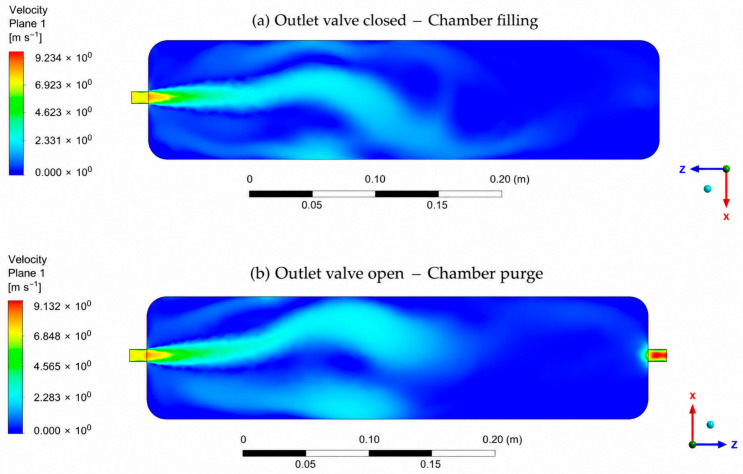
CFD simulation of the methane sensing chamber during the two operating stages of the measurement cycle. (**a**) Chamber filling stage with the outlet solenoid valve closed, illustrating the gas flow entering the sensing chamber. (**b**) Chamber purge stage with the outlet solenoid valve open, illustrating the airflow leaving the chamber after the measurement period. The color scale represents the simulated airflow velocity magnitude inside the chamber.

A new chamber geometry was developed to better accommodate integration within the vehicle while strictly preserving the calculated internal volume. In addition to packaging constraints, the internal flow distribution plays a critical role in determining the effectiveness of gas exchange. Therefore, Computational Fluid Dynamics (CFD) simulations were performed using ANSYS Fluent 2021 R2 (ANSYS Inc., Canonsburg, PA, USA) [19] to analyze and optimize the proposed chamber geometry, as illustrated in Figure 3. The simulations followed a standard workflow, including geometry definition, boundary condition setup, mesh generation, convergence analysis, and solution evaluation. The inlet mass flow rate was derived from the pump capacity and air density, ensuring realistic operating conditions representative of the final sensing platform.

Two sensing chamber geometries were investigated during the development of the proposed platform. Chamber Design 1 corresponds to the initial analytical design obtained from the theoretical chamber volume calculation. Chamber Design 2 is the final optimized geometry derived from CFD simulations. Only Chamber Design 2 was adopted for the laboratory calibration and all field experiments presented in this work.

**Figure 4:**
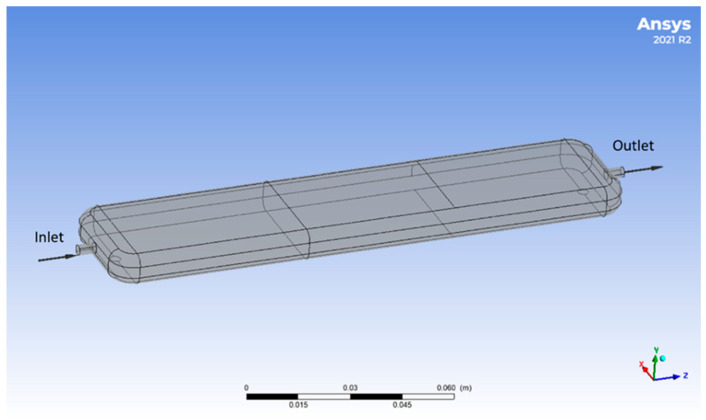
Evolution of the sensing chamber geometry from the initial analytical design to the CFD-optimized design adopted in the final sensing platform.

The optimized chamber design not only improves gas exchange efficiency but also directly impacts the temporal characteristics of the measured signal. By ensuring a more uniform and consistent flow distribution within the sensing chamber, the proposed geometry reduces spatial concentration gradients and minimizes the presence of stagnant regions, resulting in a more representative and stable signal at each sampling instant. CFD results obtained with ANSYS Fluent 2021 R2 demonstrated a more homogeneous flow pattern throughout the chamber volume, supporting the selection of the final geometry adopted in the sensing platform. The new chamber design is illustrated in Figure 4.

The CFD simulations were conducted under steady-state conditions using a pressure-based solver, with air modeled as an incompressible fluid at ambient temperature and pressure.

## 8. Electronic Systems

**Figure 5:**
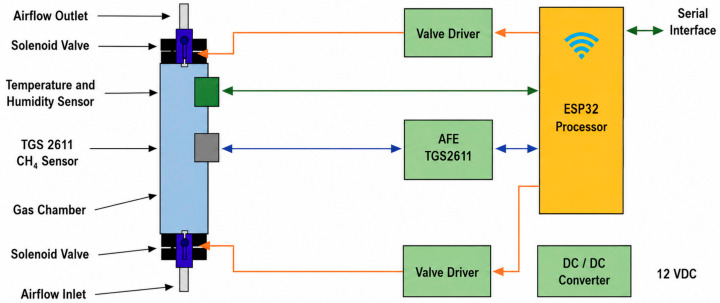
Electronic architecture of the proposed methane sensing module, including the analog front-end, environmental sensors, ESP32 microcontroller, wireless communication interfaces, and power management circuitry.

The electronic system architecture is illustrated in Figure 5.

The analog front end (AFE) module is responsible for conditioning and calibrating the analog signals generated by the TGS2611-C00 sensors, which are used for methane concentration measurement. The analog front-end (AFE) was specifically designed to acquire the electrical response of the TGS2611-C00 metal-oxide semiconductor (MOS) methane sensor by independently measuring the sensor voltage and excitation current, allowing the real-time calculation of the sensing resistance (Rs), which is the primary parameter used for methane concentration estimation.

**Figure 6:**
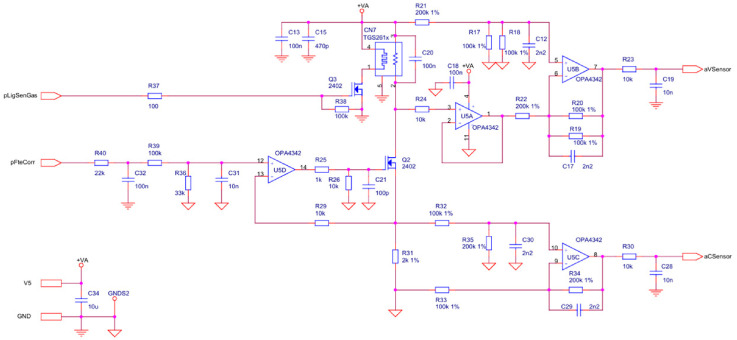
Analog front-end employed for the TGS2611−C00 methane sensor. The circuit comprises a heater driver, programmable constant-current excitation source, voltage conditioning stage, current conditioning stage, low pass filters, and analog outputs connected to the embedded controller ADCs.

Figure 6 illustrates the complete analog front-end employed in the proposed sensing platform. The circuit is composed of four functional blocks:

- Heater driver;
- Constant-current excitation source;
- Voltage conditioning circuit;
- Current conditioning circuit.

The heater driver controls the integrated sensor heater through transistor Q3, allowing the sensing element to be energized only during the measurement cycle. The heater operates from a regulated 5 V supply, maintaining the sensing material at its nominal operating temperature while reducing unnecessary power consumption outside the acquisition period.

The sensing element is excited by a programmable constant-current source implemented with operational amplifier OPA4342 (Texas Instruments, Dallas, TX, USA) U5D, transistor Q2, and the associated precision resistor network. The excitation current is generated from a DAC output provided by the ESP32 microcontroller (Espressif Systems, Shanghai, China) [20], filtered by a low-pass network, and converted into a controlled current through the sensing resistor Rs. Maintaining a constant excitation current allows the sensor resistance to be calculated directly from the measured voltage while minimizing errors associated with supply voltage variations.

The voltage developed across the sensing resistor is buffered and amplified by two precision operational amplifiers OPA4342 (U5A and U5B), producing the analog output signal aVSensor. Similarly, the voltage proportional to the excitation current is conditioned by a differential amplifier OPA4342 (U5C), generating the output aCSensor. Both conditioned analog signals are filtered using low-pass RC networks to reduce electrical noise before acquisition by the microcontroller analog-to-digital converters (ADCs).

The sensing resistance is calculated from the measured voltage and current according to Ohm’s law,

(4) $$R_{s}=\frac{V_{RS}\;}{I_{RS}}$$

where $V_{RS}\;$ is the voltage across the sensing element and $I_{RS}\;$ is the excitation current.

Considering the gain of the voltage and current conditioning stages, the sensing resistance is obtained from the digitized signals as

(5) $$R_{s}=\frac{aVSensor\cdot G_{I}\cdot R_{31}}{aCSensor\cdot G_{V}}$$

where $G_{V}$ and $G_{I}$ are the gains of the voltage and current conditioning circuits, respectively, and $R_{31}$ is the precision current-sensing resistor. This approach enables the direct estimation of the instantaneous sensor resistance independently of supply voltage fluctuations or component tolerances.

The calculated resistance is subsequently compensated for temperature and relative humidity using the calibration model presented in Section 9. The compensated resistance is then converted into methane concentration through the nonlinear calibration function obtained experimentally during laboratory calibration.

Humidity and temperature inside the sampling chambers are monitored by AHT10 temperature and relative humidity sensor (Guangzhou Aosong Electronics Co., Ltd., Guangzhou, China) [21], which communicate with the processing unit via the I^2^C (Inter Integrated Circuit) interface. These parameters are essential for compensating sensor variability and ensuring measurement reliability.

The electronic actuation subsystem consists of valve and pump drivers responsible for controlling the solenoid valves and the flow pump, enabling precise regulation of the gas sampling process.

**Figure 7:**
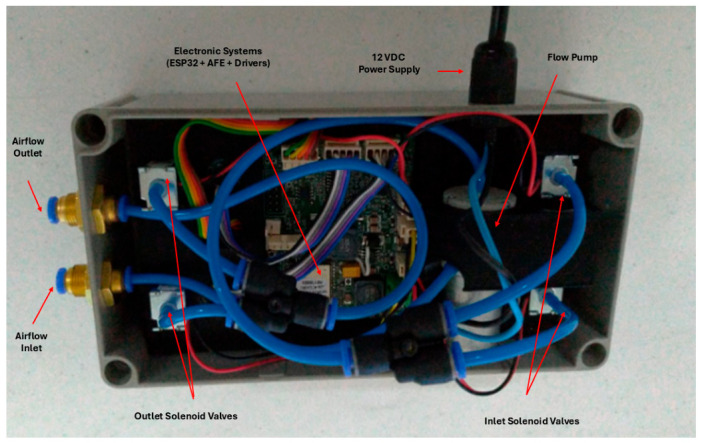
Experimental Prototype.

The processing unit is based on the ESP32 microcontroller, which integrates a dual-core 32-bit Tensilica Xtensa LX6 processor operating at up to 240 MHz, embedded Wi-Fi and Bluetooth communication interfaces, analog-to-digital converters (ADC), digital input/output peripherals, and low-power operation modes. The processing unit performs multiple functions, including sensor data acquisition, execution of calibration algorithms, system control, and data management. In addition, measurement data are transmitted through the integrated Bluetooth and Wi-Fi communication interfaces, enabling real-time connectivity with the mobile application and cloud-based infrastructure. The operation of the valves and flow pump is governed by a control model designed to account for key system parameters, including sensor response time, vehicle speed, chamber volume, and pump flow rate. This coordinated control strategy ensures proper synchronization between gas sampling and sensor dynamics. Temperature and humidity monitoring inside the chambers, provided by the AHT10 sensors, also contributes to protecting the TGS2611 sensors from adverse operating conditions, such as excessive humidity and temperature variations, which could compromise sensor performance and long-term stability. Figure 7 illustrates the experimental prototype of the mobile methane sensing system.

The smartphone was employed as a communication gateway to simplify prototype development by integrating GNSS positioning, Bluetooth communication, cellular connectivity, local storage, cloud communication, and user interface into a single commercially available device. This approach accelerated the experimental validation of the proposed sensing platform. Although a dedicated embedded gateway would provide lower power consumption and a more compact implementation, the smartphone-based architecture offered a flexible and rapidly deployable solution for large-scale field experiments.

The electronic controller communicates with the smartphone via Bluetooth or Wi-Fi, depending on the distance between the sensing module and the mobile device within the vehicle. The smartphone, through a dedicated application specifically developed for this purpose, associate geolocation data with the measured methane concentrations and transmits the indexed data to a cloud-based infrastructure for storage and further analysis [6,22].

## 9. Sensor Calibration

**Figure 8:**
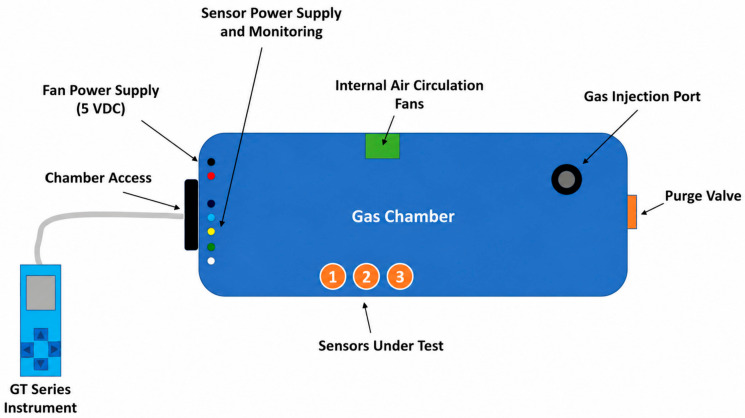
Chamber Calibration.

The calibration procedure was carried out using a laboratory calibration chamber specifically developed for controlled methane exposure. The calibration system consisted of a 240 L sealed chamber equipped with a purge outlet, a gas injection port, an internal air circulation fan, and a connection port for the reference methane analyzer (GT Series), as illustrated in Figure 8.

It should be noted that two different reference instruments were employed in this work. The GT Series portable methane analyzer was used exclusively during the laboratory calibration procedure to establish the calibration model under controlled conditions. In contrast, the Protheo infrared methane analyzer was used only during the field campaigns as the reference instrument for comparison with the proposed mobile sensing platform.

The internal circulation fan continuously homogenized the gas mixture inside the chamber, ensuring a uniform methane concentration throughout the calibration volume. The chamber also included six electrical feedthrough connectors, allowing simultaneous power supply and data acquisition from up to three sensing modules during each calibration experiment.

Each sensing module executed embedded firmware responsible for periodically acquiring methane, temperature, and relative humidity measurements, actuating the dual sampling chamber solenoid valves according to the measurement cycle, and transmitting all sensor data via Bluetooth to a computer for real-time logging and subsequent analysis.

Before each calibration experiment, the chamber was purged to eliminate residual methane from previous tests. Purging was performed using a 1000 W vacuum blower (Electrolux, São Carlos, Brazil ) connected to the chamber exhaust port and operated continuously for 30 min. After the purge procedure, the sensing modules were installed inside the chamber, which was then sealed. The reference GT Series analyzer remained connected throughout the experiment to continuously monitor the methane concentration inside the chamber.

Following system initialization, the sensing modules performed a 1 min warm-up period, after which the internal sampling pump was activated and the alternating dual-chamber measurement cycle was initiated. Raw methane sensor resistance, calculated methane concentration, chamber temperature, and relative humidity were continuously transmitted to the data acquisition computer.

The sensor baseline (zero point) was established after the methane readings reached a stable condition while the reference GT Series analyzer indicated zero methane concentration. Once the baseline had stabilized, known methane concentrations were generated by injecting calibrated gas volumes into the 240 L chamber. The theoretical methane concentration was calculated according to:

(6) $$C_{calc}=\frac{1000\times V_{inj}}{240}$$

where $V_{inj}$ is the injected methane volume (mL) and $C_{calc}$ is the theoretical methane concentration (ppm).

After each gas injection, the chamber was allowed to homogenize before recording the methane concentration measured by the GT Series analyzer together with the resistance values of the MOS sensors installed in Chambers 1 and 2. New gas injections were performed at 10 min intervals, producing progressively higher methane concentrations. Table 2 summarizes the injected methane volumes, the calculated methane concentrations, the concentrations measured by the reference instrument, and the corresponding resistance values obtained from both sensing chambers.

During the calibration experiments, the sensor conditions remained relatively stable, with temperatures ranging from 41 °C to 44 °C and relative humidity between 30% and 33%, as recorded by the integrated environmental sensors. These measurements were simultaneously logged during all calibration procedures and were used to verify the stability of the experimental conditions.

Although the environmental conditions inside the calibration chamber remained relatively stable throughout the experiments, the temperature and relative humidity measurements were incorporated into the calibration algorithm to compensate for the environmental dependence of the MOS sensing element. The TGS2611-C00 sensor exhibits variations in sensing resistance as a function of ambient temperature and humidity due to changes in the surface adsorption processes occurring on the tin dioxide sensing layer. Therefore, before applying the methane concentration calibration function, the measured sensor resistance was computationally normalized to the reference environmental condition (20 °C and 65% relative humidity) using a compensation factor derived from the manufacturer’s temperature and humidity characteristic curves.

The compensated sensor resistance was calculated as

(7) $$R_{s,corr}=\frac{R_{s}}{K(T,RH)}$$

where $R_{s,corr}\;$ is the temperature and humidity-compensated sensor resistance, $R_{s}\;$ is the measured sensor resistance, and K(T,RH) is the environmental compensation factor obtained by bilinear interpolation of the manufacturer’s temperature and relative humidity characteristic curves. The interpolation was performed using the four nearest operating points extracted from the typical temperature–humidity characteristic graph provided in the TGS2611-C00 datasheet, yielding a continuous compensation factor over the entire operating range. The compensation factor is normalized such that K=1  at the reference condition of 20 °C and 65% relative humidity, as specified in the TGS2611-C00 datasheet [10]. This compensation minimizes the influence of environmental variations on the sensor baseline and sensitivity, allowing the subsequent methane concentration estimation to be based primarily on the gas concentration rather than on changes in ambient conditions. Although the laboratory calibration was performed under nearly constant environmental conditions, the compensation algorithm was implemented in the embedded firmware to ensure accurate operation during outdoor measurements, where temperature and relative humidity may vary significantly.

**Figure 9:**
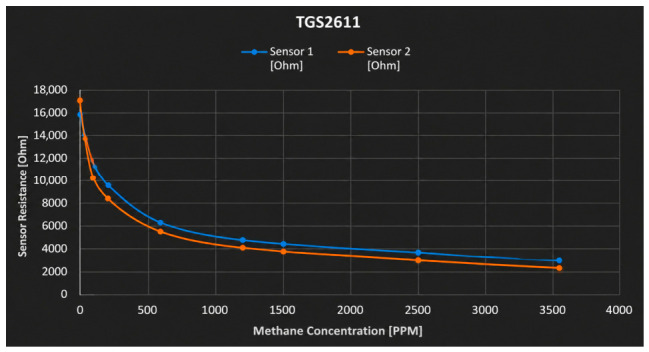
Experimental response of the TGS2611 methane sensors as a function of methane concentration.

Figure 9 presents the response of the two TGS2611 methane sensors as a function of methane concentration, using the calibration data summarized in Table 2. The graph was constructed from the experimental measurements obtained during the laboratory calibration procedure, in which the methane concentration was determined using the reference GT Series analyzer and correlated with the electrical resistance of each sensor. As expected for metal-oxide semiconductor (MOS) sensors, the sensor resistance decreases nonlinearly as the methane concentration increases. A rapid reduction in resistance is observed at low methane concentrations, followed by a more gradual decrease at higher concentrations, resulting in the characteristic inverse response of the TGS2611 sensor. The close agreement between the responses of Sensors 1 and 2 demonstrates good repeatability and consistency between sensing elements, supporting the subsequent development of the calibration functions implemented in the embedded firmware.

**Figure 10:**
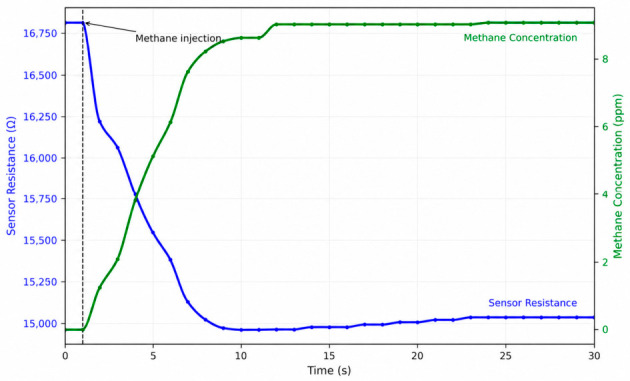
Experimental transient response of the TGS2611-C00 sensor following the injection of 9 ppm methane into the calibration chamber. The raw sensor resistance (left axis) and the methane concentration measured by the GT Series reference analyzer (right axis) are presented as a function of time. The curves were generated from the experimental data using shape-preserving interpolation to improve visualization, while the markers represent the original measured data points. The figure illustrates the primary sensor response before temperature–humidity compensation, moving-average filtering, and nonlinear calibration.

Figure 10 illustrates a representative transient response of the TGS2611-C00 sensor obtained during the laboratory calibration procedure following the injection of 9 ppm methane into the calibration chamber. The figure presents the raw sensor resistance acquired directly from the analog front-end before the application of temperature–humidity compensation, moving-average filtering, and the nonlinear calibration algorithm. As expected for a metal-oxide semiconductor sensor, the sensing resistance decreases rapidly after methane exposure and subsequently approaches a stable equilibrium value. This primary experimental response illustrates the raw data used by the embedded firmware to estimate methane concentration.

The calibration function was obtained by nonlinear regression using the compensated sensor resistance dataset summarized in Table 2. Initially, the measured sensor resistance was corrected for temperature and relative humidity effects using the environmental compensation model described above. The compensated resistance values were then correlated with the methane concentrations measured by the certified GT Series reference analyzer. Because the TGS2611 sensor exhibits a nonlinear response over the investigated concentration range, as illustrated in Figure 9, the experimental data were divided into two concentration intervals, and each interval was independently fitted using a three-parameter rational function.

(8) $$C=M+\frac{A}{R_{s,corr}-R_{0}}$$

The proposed calibration methodology can be interpreted as a sequential environmental compensation model followed by nonlinear methane calibration, in contrast to multivariable regression approaches where temperature and relative humidity are incorporated directly into the regression model.

Where C represents the methane concentration (ppm), $R_{s,corr}\;$ is the temperature and humidity compensated resistance (Ω), and M, A, and $R_{0}$ are fitting parameters. The coefficients were estimated by nonlinear least-squares regression using the experimental calibration dataset, selecting the model that minimized the residual fitting error.

**Figure 11:**
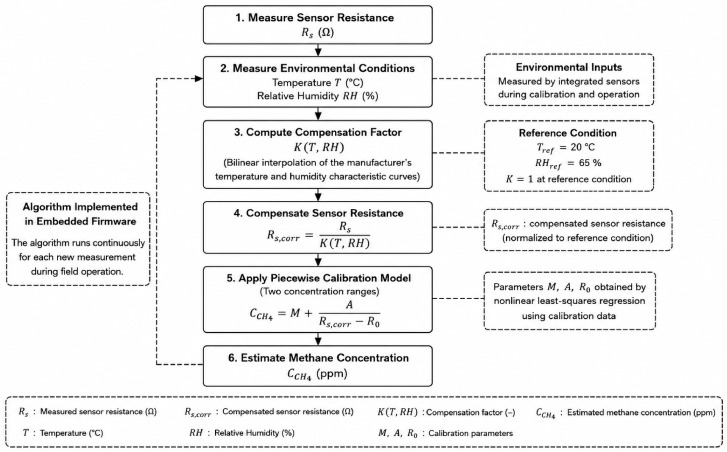
Flowchart of the methane concentration estimation algorithm implemented in the embedded firmware.

The resulting calibration equations were subsequently implemented in the embedded firmware of the sensing platform. Figure 11 illustrates the flowchart of the methane concentration estimation algorithm implemented in the firmware. During operation, the firmware continuously measures the electrical resistance of the TGS2611 sensor together with the ambient temperature and relative humidity. The measured sensor resistance is first compensated for environmental effects using the temperature–humidity compensation model described in Equation (7). The compensated resistance is then used to automatically select the appropriate piecewise calibration equation according to the corresponding resistance interval, allowing the methane concentration to be calculated in real time. This approach minimizes the influence of environmental variations on the sensor response while significantly improving the conversion accuracy over the entire measurement range, without compromising the computational efficiency required for embedded implementation.

## 10. Systems Operation and Data Analysis

**Figure 12:**
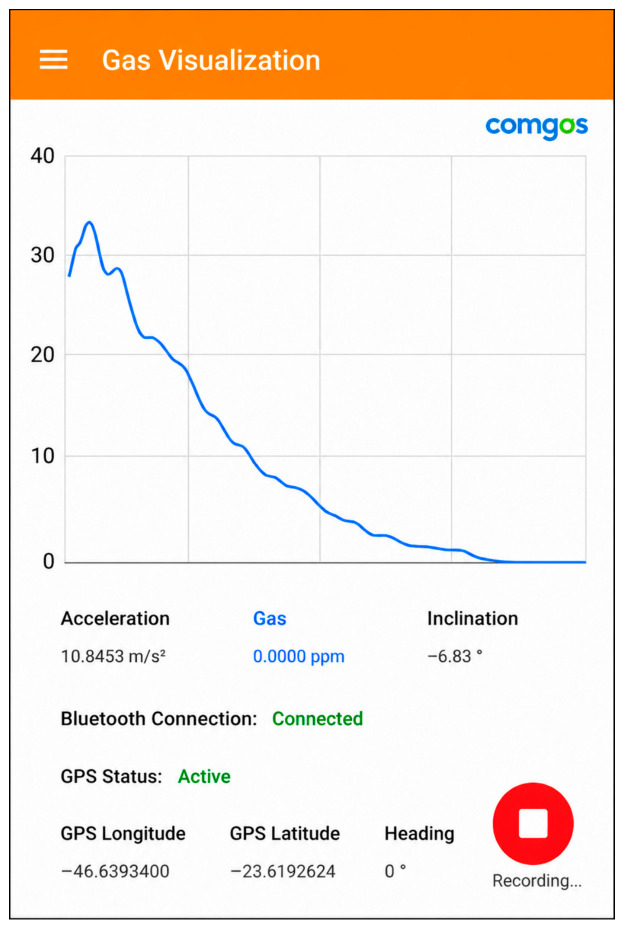
Monitoring Application.

A dedicated mobile application was developed for system monitoring and control, enabling communication, data indexing, alarm management, and visual tracking [23]. The application provides a dashboard for real time measurement visualization and issues acoustic alarms in case of elevated methane concentrations. The application interface is depicted in Figure 12.

When the vehicle is starting, the driver connects the smartphone to the sensor via Bluetooth using the monitoring application illustrated by Figure 12. The sensor first enters the exhaust mode, purging the chambers for subsequent measurements. Simultaneously, the TGS2611 sensor begins its warmup phase, which lasts approximately one minute.

Although the manufacturer recommends a longer stabilization period after power-up, preliminary laboratory experiments demonstrated that the sensor response reached a sufficiently stable and repeatable condition after approximately 60 s under the operating conditions adopted in this work. Therefore, a 1 min warm-up period was selected as a compromise between measurement stability and the operational requirements of mobile monitoring.

**Figure 13:**
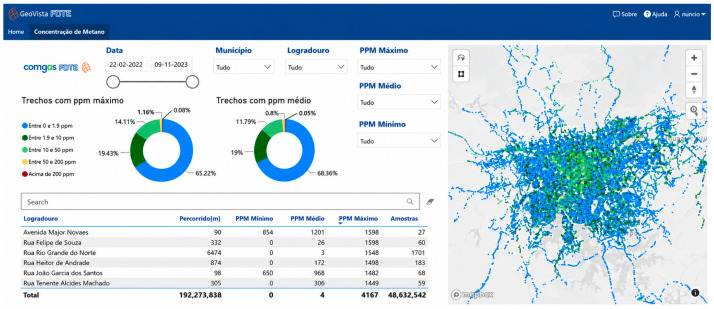
Monitoring Dashboard.

After this warmup, the exhaust process is terminated, and methane measurement cycles commence, alternating readings between the gas chambers. Methane concentration data are transmitted to the smartphone via Bluetooth, where the application appends metadata including latitude, longitude, date, time, and sensor identifier. The complete dataset is then uploaded to a cloud-based database for further processing. Concentration levels at different locations are monitored through a dedicated control dashboard. Sensors were deployed on 16 vehicles for testing and data collection, as illustrated in Figure 13.

**Figure 14:**
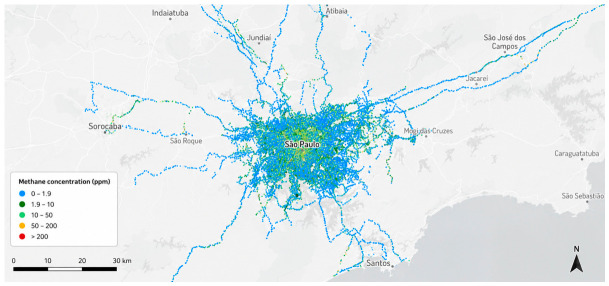
Monitored Roads Map.

Over a 20-month period, the 16 vehicles traveled approximately 192,274 km across the metropolitan region of São Paulo, collecting 48,632,542 samples, with an average spatial resolution of one sample every 3.95 m. The coverage of the monitored road network is shown in Figure 14.

## 11. Comparative Analysis and Validation of the TGS2611 Sensor

**Figure 15:**
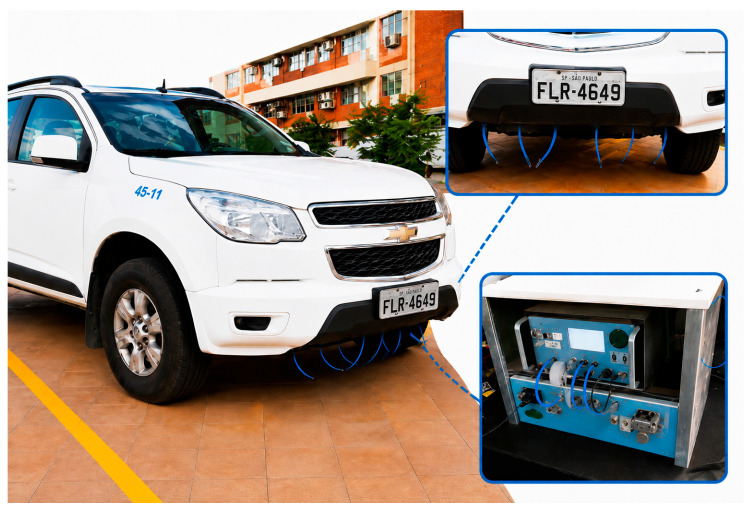
Vehicle equipped with Protheo Huberg and TGS2611 methane sensing systems.

In the vehicle shown in Figure 15, where the Protheo Huberg sensor system is employed for test measurements, the proposed methane sensing system based on the TGS2611 sensor was installed. The Protheo Huberg unit operates by collecting measurement data and storing it locally in onboard memory, which are subsequently downloaded for offline analysis.

In contrast, the TGS2611-based system transmits measurement data in real time, relying on a dedicated communication interface and exhibiting a strong dependence on the availability and quality of local 3G/4G network services.

**Figure 16:**
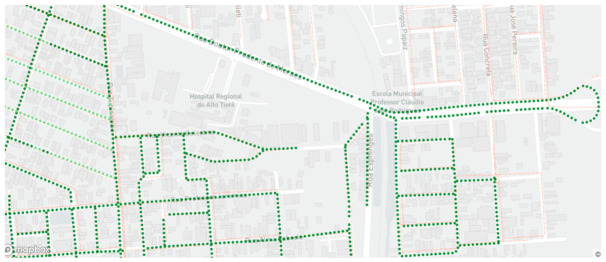
TGS2611 Data Map.

**Figure 17:**
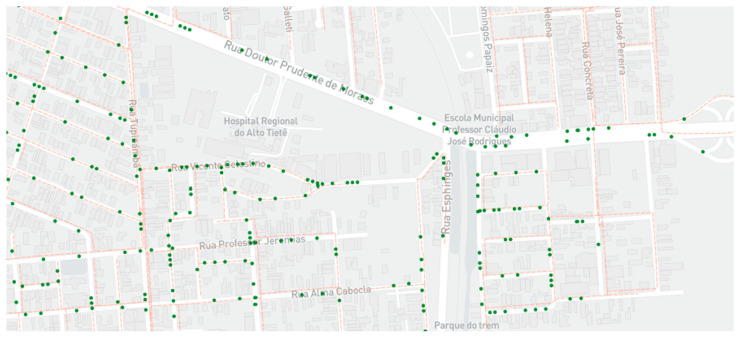
Protheo Huberg Data Map.

A comparative analysis was performed between the TGS2611-based system and the Protheo Huberg reference instrument using the spatial methane concentration maps shown in Figure 16 and Figure 17. The objective was to evaluate the ability of the low cost TGS2611 sensor to represent methane concentration patterns under real vehicular conditions.

The Protheo Huberg system serves as a reliable reference for methane monitoring, employing infrared sensing technology and a well-established measurement methodology. A comparison of the spatial distribution maps reveals that the TGS2611-based platform provided a denser spatial representation in the analyzed dataset. This improved spatial coverage is associated with the sampling strategy adopted by the proposed system, resulting in a denser representation of methane concentration patterns along the monitored routes.

Although the two systems differ in sensing principles, sampling strategies, and data acquisition architectures, both datasets exhibit comparable spatial methane distribution patterns. Areas identified by the Protheo Huberg system as presenting elevated methane concentrations are also detected by the TGS2611 platform, indicating consistency in the identification of concentration zones and potential emission hotspots. The visual agreement observed in the concentration maps demonstrates that both systems capture the same large-scale spatial features despite differences in temporal sampling and measurement density.

A direct point-to-point comparison between individual measurements resulted in limited statistical agreement, with moderate overall class concordance and low Cohen’s Kappa values. These results are expected given the absence of temporal synchronization between datasets, the highly dynamic nature of urban methane concentrations, and the distinct sampling methodologies employed by each system. Consequently, point-by-point correspondence does not represent the most appropriate validation approach for mobile methane monitoring applications.

Instead, the comparison of spatial concentration patterns, hotspot localization, and concentration class distributions provides a more representative assessment of system performance. Under this framework, the TGS2611-based platform demonstrates a consistent ability to identify regions of elevated methane concentration and reproduce the principal spatial trends observed by the reference system.

Therefore, the results support the applicability of the proposed TGS2611-based mobile sensing platform for large-scale urban methane monitoring. Beyond reproducing the main spatial patterns identified by the infrared reference system, the platform provides increased measurement density, broader geographic coverage, and enhanced capability for identifying methane concentration zones across extensive urban areas. These characteristics make the proposed solution suitable for scalable environmental monitoring and methane screening applications where spatial trend identification is prioritized over precise point-to-point concentration matching.

It should be noted that the Protheo Huberg reference measurements were available only for selected urban routes. Therefore, the comparison presented in Figure 16 and Figure 17 is restricted to the common road segments surveyed by both sensing systems and does not represent the entire monitoring area covered by the proposed platform.

To complement the qualitative comparison based on spatial concentration maps, a quantitative analysis was performed by associating each reference measurement point with the nearest TGS2611 measurement within predefined maximum distances of 50 m and 100 m. The evaluation considered concentration class agreement, Cohen’s Kappa coefficient, and event detection metrics. The results are summarized in Table 3. Low Kappa values should not be interpreted as a failure of spatial hotspot detection, but rather as a consequence of comparing non-synchronized mobile datasets with different sensing technologies and acquisition strategies.

The class distribution shown in Table 4 indicates that the TGS2611-based system detected a broader range of methane concentration levels than the Protheo Huberg dataset. This difference may be associated with the distinct sensing principles, acquisition strategies, temporal sampling conditions, and spatial coverage of each system. Therefore, the comparison reinforces that the validation should be interpreted in terms of spatial concentration patterns and hotspot identification rather than direct equivalence between individual concentration values.

## 12. Results

The field measurements obtained with the proposed sensing platform were compared with those acquired by the Protheo Compact reference analyzer described in Section 2 Reference Methane Analyzer.

**Figure 18:**
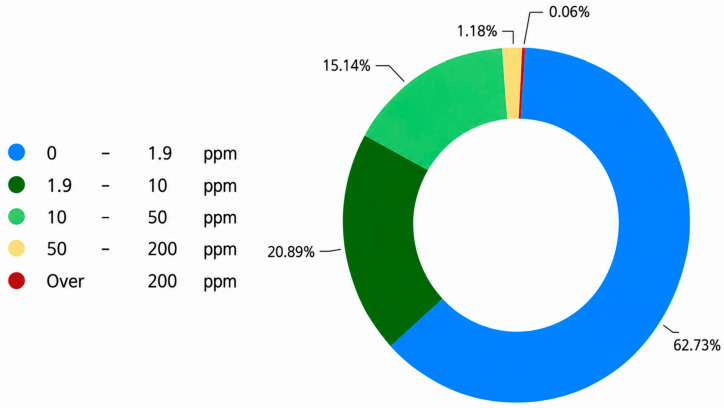
São Paulo City Monitored Roads Map.

During field tests, several areas with elevated methane concentrations were identified. Figure 18 presents the graphic results for the municipality of São Paulo, where approximately 145,823 km were covered. Among the monitored roads, 62.73% exhibited concentrations between 0 and 1.90 ppm values compatible with typical atmospheric methane background concentrations reported by NOAA [2].

**Figure 19:**
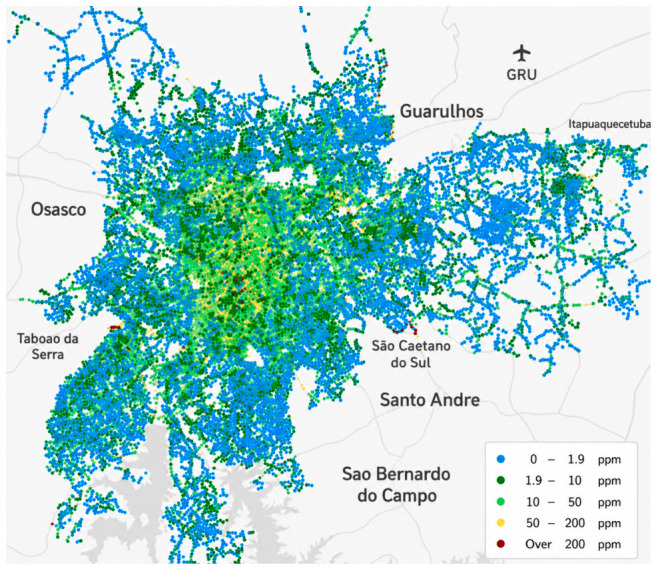
Methane Concentration Map.

The data collected by sensors mounted on different vehicles traveling through the same areas at different times revealed consistent concentration levels. In the graph shown in Figure 18, average concentrations of 1.0 ppm were observed over approximately 123,700 km of monitored roads. Conversely, Figure 19 highlights a central area with an average concentration of 17 ppm, corresponding to 22,085 km of monitored road segments.

## 13. Discussion

The results demonstrate the effectiveness of the proposed mobile sensing approach for identifying spatial methane concentration patterns in urban environments. In São Paulo, most measurements (62.73%) fall within background levels (0–1.90 ppm), while 37.27% exceeded the atmospheric background concentration adopted in this study (1.9 ppm), confirming that methane emissions are spatially heterogeneous and concentrated in specific regions.

The identification of areas with higher average methane concentrations, such as the central region with values reaching 17 ppm, indicates the presence of persistent emission sources that can be detected through repeated mobile measurements. The consistency of measurements obtained from different vehicles operating over the same road segments further demonstrates the capability of the proposed system to capture stable spatial concentration trends over time.

Comparison with the Protheo Huberg reference system demonstrates that the proposed TGS2611-based sensing platform is capable of reproducing the principal spatial patterns of methane distribution observed in urban environments. Although the direct point-to-point comparison between individual measurements resulted in limited statistical agreement, the spatial distribution maps revealed a consistent correspondence in the identification of concentration zones and potential methane hotspots. Similar findings have been reported in previous studies employing mobile sensing platforms and low-cost MOS sensors for large-scale environmental monitoring applications.

Differences in the peak methane concentrations reported by the two systems should not be interpreted as a limitation of either instrument. Instead, they primarily reflect differences in sensing technology, gas sampling geometry, sensor dynamics, and acquisition methodology, which influence the instantaneous measurement of narrow methane plumes during vehicle motion.

The observed differences should also be interpreted considering the distinct sensing principles of the two instruments. The Protheo Huberg analyzer is based on infrared absorption and provides highly selective methane measurements, whereas the proposed sensing platform employs a TGS2611-C00 metal-oxide semiconductor sensor whose response may be influenced by other reducing gases commonly present in urban environments, including carbon monoxide, hydrogen, alcohol vapors, and hydrocarbons. Consequently, under certain operating conditions, the MOS sensor may overestimate methane concentration due to cross-sensitivity. This limitation is inherent to MOS sensing technology and has been explicitly addressed in the present work through laboratory calibration, temperature–humidity compensation, and repeated spatial measurements. Therefore, the objective of the proposed platform is not to replace high-selectivity infrared analyzers, but to provide a scalable and cost-effective screening tool capable of identifying persistent methane hotspots for subsequent investigation using reference-grade instruments.

The results indicate that spatial pattern analysis provides a more appropriate validation framework than individual measurement matching when comparing mobile methane monitoring systems operating under different sampling methodologies and acquisition conditions. The observed agreement in concentration zones, together with the comparable distribution of methane concentration classes across the monitored area, supports the capability of the proposed system to identify regions associated with elevated methane levels. Although the proposed approach does not provide the same metrological accuracy and selectivity as high-end infrared-based instruments, it offers important advantages in terms of deployment simplicity, compactness, scalability, and cost-effectiveness.

Although the proposed approach does not provide the same metrological accuracy and selectivity as high-end infrared-based analyzers, it demonstrated the capability to reproduce the principal spatial methane concentration patterns observed by the reference system. The main contribution of the proposed platform is therefore not to replace high-precision infrared analyzers, but to provide a scalable and cost-effective solution for large-scale urban methane hotspot screening through repeated mobile measurements.

Its low cost, compact architecture, and ease of deployment enable the implementation of dense mobile sensing networks that would be economically difficult to achieve using only reference-grade infrared instruments.

These characteristics are particularly relevant for continuous monitoring over extensive urban regions, where the large-scale deployment of high-cost reference instruments may be economically impractical.

Some limitations of the system should also be considered. Environmental factors such as temperature and humidity can influence the MOS sensor response, although these effects were mitigated in the proposed system through the environmental compensation model described in Section 9. Sensor drift and cross-sensitivity to other reducing gases may also affect long-term measurement stability. In particular, the TGS2611-C00 exhibits negligible sensitivity to carbon dioxide (CO_2_), whereas limited cross-sensitivity may occur in the presence of carbon monoxide (CO) and other reducing gases, which is an inherent characteristic of metal-oxide semiconductor (MOS) sensing technology.

Although the TGS2611-C00 exhibits limited cross-sensitivity to certain reducing gases commonly present in urban environments, including carbon monoxide, hydrogen, alcohol vapors, and hydrocarbons, despite this limitation, the sensor remains suitable for large-scale methane hotspot screening. The proposed sensing platform is intended to identify persistent spatial methane concentration anomalies rather than to perform chemical source attribution. The comparison with the reference infrared methane analyzer demonstrated that both sensing systems identified similar methane concentration patterns despite their different sensing principles. Consequently, the influence of occasional cross-interference is minimized through laboratory calibration, temperature–humidity compensation, repeated measurements, and the identification of persistent spatial concentration patterns instead of isolated sensor readings.

If the elevated methane concentrations observed during the monitoring campaign were predominantly false-positive responses caused by sensor cross-sensitivity, one would expect a random spatial distribution of the detected anomalies. Instead, the observed methane hotspots were repeatedly identified at the same locations by different vehicles over a 20-month monitoring campaign comprising more than 48 million measurements. This persistence strongly suggests that the observed spatial patterns represent stable environmental features rather than random sensor artifacts or transient interference. Future developments may incorporate complementary sensing technologies to further improve discrimination between methane and other reducing gases in complex urban environments.

Future work will also include controlled laboratory experiments comparing the proposed platform, the Protheo Huberg analyzer, and an independent reference-grade methane analyzer under known methane concentrations and controlled concentrations of potential interfering gases. In addition, alternative MOS sensors, including the TGS2611-E00, will be evaluated to investigate possible improvements in methane selectivity under complex urban atmospheric conditions.

In addition, the use of a 30 s moving-average processing window may reduce sensitivity to short-duration localized methane peaks, particularly at higher vehicle speeds. However, this strategy improves measurement robustness by reducing transient fluctuations and enhancing the repeatability of spatial concentration patterns. Repeated measurements collected along the same routes further contribute to the reliability of the generated concentration maps.

An example of a persistent methane hotspot can be observed in the Taboão da Serra region (Figure 19), located in the southwestern metropolitan area of São Paulo. This region is characterized by dense urbanization, intense vehicular traffic, aging sewer infrastructure, and several canalized streams and drainage channels. Although the present study was not designed to identify the specific origin of each methane plume, repeated measurements collected by different vehicles consistently detected elevated methane concentrations in this area. These observations suggest the presence of persistent methane emission sources rather than isolated transient events, highlighting the usefulness of the proposed sensing platform for identifying regions that warrant further investigation using high-selectivity analytical instruments.

In a broader context, the proposed mobile sensing architecture represents a promising solution for smart-city environmental monitoring applications. The integration of low-cost sensing technology, vehicular mobility, wireless communication, cloud-based infrastructure, and geospatial data processing enables scalable methane monitoring strategies capable of supporting emission mapping, infrastructure inspection, leak screening, and urban environmental assessment. The results demonstrate that low-cost mobile sensing platforms can provide valuable spatial information for large-scale methane surveillance, complementing conventional high-precision monitoring systems.

An additional practical aspect concerns the selection of the carrier platform for large-scale mobile methane monitoring. In the present study, the sensing modules were deployed on a combination of ride-hailing vehicles operating under the Uber platform, vehicles owned by the research team, and test vehicles provided by the local natural gas distribution company (Comgás, São Paulo, Brazil). This approach enabled extensive spatial coverage while avoiding the costs associated with dedicated monitoring fleets. The objective of this work was to evaluate the feasibility of the proposed sensing architecture rather than to assess the environmental footprint of the carrier vehicles. Therefore, exhaust emissions from the participating vehicles were not quantified, since the monitoring campaign involved vehicles from different manufacturers, models, engine technologies, and operating conditions.

From a long-term perspective, the environmental impact of the carrier platform should be considered when designing future mobile sensing networks. As low-power gas sensing technologies continue to evolve, the proposed architecture could be integrated into electric vehicles or even personal mobile devices, such as smartphones and wearable devices, which already provide positioning, wireless communication, processing capability, and cloud connectivity. Such developments would enable large-scale distributed environmental monitoring while further reducing the environmental impact associated with the sensing platform itself.

## 14. Conclusions

This work presented the development and large-scale evaluation of a vehicular IoT-based methane monitoring system integrating a TGS2611 MOS sensor, dual-chamber gas sampling architecture, embedded processing, wireless communication, and cloud-based data analysis. The proposed platform was designed as a low-cost and scalable solution for urban methane monitoring, enabling continuous data acquisition under real-world operating conditions.

Field experiments conducted in the metropolitan region of São Paulo demonstrated the capability of the system to identify spatial methane concentration patterns across extensive urban areas, covering approximately 145,823 km of monitored roads. The collected data revealed both background methane levels and localized regions with elevated concentrations, indicating the presence of persistent emission sources and spatially heterogeneous methane distributions.

The comparative analysis with the Protheo Huberg infrared-based reference system demonstrated that the proposed TGS2611-based platform can successfully reproduce the principal spatial patterns of methane distribution observed in the monitored area. Although direct point-to-point comparisons resulted in limited statistical agreement due to differences in sampling methodologies, temporal synchronization, and the inherently dynamic nature of urban methane concentrations, both systems consistently identified similar concentration zones and potential emission hotspots. Furthermore, the TGS2611-based platform achieved a higher density of measurement points and a more homogeneous spatial coverage, providing a detailed representation of methane concentration patterns along the surveyed routes.

The results indicate that spatial pattern analysis, hotspot localization, and concentration class distribution provide a more representative framework for validating mobile methane monitoring systems than direct comparison of individual measurements. Within this context, the proposed platform demonstrated a consistent ability to identify regions of elevated methane concentration and reproduce the main spatial trends detected by the reference system.

Although the system remains subject to limitations associated with MOS sensing technology, including environmental sensitivity, cross-sensitivity, sensor drift, and reduced capability to capture short-duration concentration peaks, the overall results demonstrate that low-cost MOS-based sensing platforms can provide practical, reliable, and scalable solutions for large-scale urban methane screening and environmental monitoring applications.

Future work will focus on improving calibration methodologies, implementing environmental compensation techniques, increasing temporal and spatial resolution, and exploring advanced geospatial and machine-learning-based data processing approaches for enhanced concentration estimation, hotspot detection, and emission source characterization. The proposed architecture represents a promising tool for continuous urban environmental monitoring and may contribute to decision-making processes related to methane emission management, infrastructure assessment, and urban sustainability initiatives.

## Figures and Tables

**Table 1:** Comparison of the main characteristics of the MOS (TGS2611) and NDIR (INIR) methane sensing technologies used in this work.

Parameter	MOS (TGS2611)	NDIR (INIR)	Remarks
Measurement principle	Metal Oxide Semiconductor	Infrared absorption spectroscopy	Different sensing principles.
Target gas	CH_4_ (Cross-sensitive to other reducing gases)	CH_4_ only	NDIR provides higher gas selectivity.
Response time (T_90_)	≈30 s	≈2–5 s	NDIR responds significantly faster.
Measurement resolution	≈5–10 ppm (after calibration)	1 ppm	According to manufacturer specifications.
Selectivity	Moderate	Very high	MOS may respond to H_2_, CO, alcohols and hydrocarbons.
Cross-sensitivity	Moderate	Very low	MOS responds to several reducing gases, whereas NDIR is highly selective for methane.
Sensitivity to CO	Moderate	Negligible	CO may produce a measurable response in MOS sensors because it is a reducing gas, whereas NDIR methane analyzers are essentially insensitive to CO.
Sensitivity to CO_2_	Negligible	Negligible	CO_2_ has minimal influence on both technologies.
Sensitivity to H_2_	High	Negligible	H_2_ is one of the principal interferents of MOS sensors.
Sensitivity to Alcohol Vapors	Moderate–High	Negligible	Ethanol and other VOCs may influence MOS measurements.
Long-term stability	Moderate	High	MOS requires periodic recalibration.
Operating power	≈280 mW	≈1–3 W	NDIR requires an IR source and optical detector.
Sampling interval	1 s	1 s	Both systems provide continuous measurements.
Warm-up time	≈1 min (this work) *	Manufacturer specified	MOS warm-up experimentally optimized.
Ease of integration	Very high	Moderate	MOS is compact and easily integrated into embedded systems
Relative cost	Low	High	MOS technology is considerably less expensive.
Typical application	Low-cost distributed sensing	High-precision methane analysis	Technologies are complementary.
Reference used in this work	Mobile sensing platform under development	Reference analyzer for field validation	NDIR was used exclusively as the field reference instrument.
Suitability for large-scale urban monitoring	Excellent (low cost, scalable, compact)	Limited (high cost, larger size)	MOS enables dense spatial sampling, whereas NDIR provides higher metrological performance.

* Although the manufacturer recommends a longer stabilization period, laboratory experiments demonstrated that a 60 s warm-up period provides stable and repeatable measurements for the proposed mobile sensing application.

**Table 2:** Laboratory calibration results obtained using the 240 L calibration chamber.

Injected CH_4_ (mL)	Calculated CH_4_ Concentration (ppm)	GT Series Reference (ppm)	Sensor 1 Resistance (Ω)	Sensor 1 Temperature (°C)	Sensor 1 Humidity (%)	Sensor 2 Resistance (Ω)	Sensor 2 Temperature (°C)	Sensor 2 Humidity (%)
0	0.0	0	16,168	42	30	16,898	41	33
5	20.8	20	14,726	43	31	14,858	41	33
10	41.7	38	13,710	43	32	13,471	42	33
30	125.0	105	10,947	43	31	10,261	42	32
50	208.3	215	9431	42	31	8691	42	33
150	625.0	594	6351	43	32	5705	41	32
300	1250.0	1202	4804	44	32	4321	42	31
600	2500.0	2453	3657	43	31	3258	42	31
1000	4166.7	3538	2983	43	32	2622	42	31

**Table 3:** Quantitative comparison between the TGS2611-based sensing platform and the Protheo Huberg reference system using spatial matching criteria.

Metric	50 m Threshold	100 m Threshold
Matched Points	3810	3985
Overall Class Concordance (%)	56.88	54.86
Cohen’s Kappa Coefficient	−0.0008	0.0027
Precision (>1.9 ppm) (%)	0.86	1.18
Recall (>1.9 ppm) (%)	33.3	42.9
F1-Score (>1.9 ppm)	0.0168	0.0229
Mean Distance Between Matched Points (m)	31.4	58.7
Maximum Matching Distance (m)	50	100

**Table 4:** Distribution of methane concentration classes obtained by the TGS2611 and Protheo Huberg systems.

Concentration Class (ppm)	TGS2611 (%)	Protheo Huberg (%)
0–1.9	57.38	98.92
1.9–10	16.04	1.08
10–50	22.34	0.00
50–200	3.41	0.00
>200	0.81	0.00

## Data Availability

The data supporting the findings of this study are available from the corresponding author upon reasonable request. The complete dataset is not publicly available because it contains proprietary georeferenced information acquired during field campaigns conducted in collaboration with the local natural gas distribution company.

## References

[1] Turner A.J., Frankenberg C., Kort E.A. (2019). Interpreting contemporary trends in atmospheric methane. Proc. Nat. Acad. Sci. USA.

[2] National Oceanic and Atmospheric Administration (NOAA) Trends in Atmospheric Methane. https://gml.noaa.gov/ccgg/trends_ch4/.

[3] Latorre M., de Carvalho O.A., de Carvalho A.P.F., Shimabukuro Y.E. (2002). Correção Atmosférica: Conceitos e Fundamentos. Espaço Geogr..

[4] Shimanoe K., Yuasa M., Kida T., Yamazoe N. Semiconductor Gas Sensor Using Nano-Sized Oxide for High-Sensitive Detection of Environment-Related Gases. Proceedings of the 2011 IEEE Nanotechnology Materials and Devices Conference.

[5] Bretschneider T.R., Shetti K. (2015). UAV-Based Gas Pipeline Leak Detection.

[6] Ishak A.J., Mahmood S.N., Hussain A.-S.T. (2019). GSM Based Gas Leak Monitoring System. Period. Eng. Nat. Sci..

[7] Kessel T.G., Ramachandran M., Klein L.J., Nair D., Hinds N., Hamann H., Sosa N.E. Methane Leak Detection and Localization Using Wireless Sensor Networks for Remote Oil and Gas Operations. Proceedings of the 2018 IEEE SENSORS.

[8] von Fischer J.C., Cooley D., Chamberlain S., Gaylord A., Griebenow C.J., Hamburg S.P., Salo J., Schumacher R., Theobald D., Ham J. (2017). Rapid, Vehicle-Based Identification of Location and Magnitude of Urban Natural Gas Pipeline Leaks. Environ. Sci. Technol..

[9] TGS2611-C00 Datasheet. https://www.figarosensor.com/product/docs/tgs2611-c00_product%20information%28fusa%29rev01.pdf.

[10] Eugster W., Kling G.W. (2012). Performance of a Low-Cost Methane Sensor for Ambient Concentration Measurements in Preliminary Studies. Atmos. Meas. Tech..

[11] Aldhafeeri T., Tran M., Vrolyk R., Pope M., Fowler M. (2020). A Review of Methane Gas Detection Sensors: Recent Developments and Future Perspectives. Inventions.

[12] Wang W.Z., Wang Y.M., Song W.J., Li X.Q. (2017). Multiband infrared inversion for low-concentration methane monitoring in a confined dust-polluted atmosphere. Appl. Opt..

[13] Zheng C., Ye W., Sanchez N.P., Li C., Dong L., Wang Y., Griffin R.J., Tittel F.K. (2017). Development and field deployment of a mid-infrared methane sensor without pressure control using interband cascade laser absorption spectroscopy. Sens. Actuators B Chem..

[14] HGS Protheo Huberg. https://www.hgs.it/it/protheo/.

[15] INIR-ME5% Datasheet. https://www.sgxsensortech.com/uploads/f_note/ds-0229-INIR.pdf.

[16] Leonardo F., Manuel F., Laercio F., Getúlio I. Aplicação e Avaliação de um Sensor de Baixo Custo para Identificação de Perdas de Metano em Redes Urbanas de Distribuição de Gás Natural. Proceedings of the Conferência Internacional de Ambiente em Língua Portuguesa, XX Encontro REALP.

[17] Portable Gas Detector GT Series. https://www.teledynegasandflamedetection.com/en-us/gt-7-applications-portable-gas-detection.

[18] Sivathanu Y. (2005). Technology Status Report on Natural Gas Leak Detection in Pipelines.

[19] Computational Fluid Dynamics Software Simulation. https://www.ansys.com/products/fluids/ansys-fluent.

[20] ESP32-WROOM 32D Datasheet. https://documentation.espressif.com/esp32-wroom-32d_esp32-wroom-32u_datasheet_en.pdf.

[21] Aosong Electronics Co., Ltd. AHT10 Technical Manual. https://server4.eca.ir/eshop/AHT10/Aosong_AHT10_en_draft_0c.pdf.

[22] Guo K., Yang P., Guo D.H., Liu Y. Gas Leakage Monitoring with Mobile Wireless Sensor Networks. Proceedings of the 8th International Congress of Information and Communication Technology (ICICT-2019).

[23] Pandey R.C., Verma M., Sahu L.K., Deshmukh S. (2018). Internet of Things (IoT) Based Gas Leakage Monitoring and Alerting System with MQ-6 Sensor. Int. J. Creat. Res. Thoughts.

